# Preferences for and acceptability of long-acting HIV prevention products among pregnant and lactating women accessing health services in Kenya: a mixed method cross-sectional analysis

**DOI:** 10.1186/s12879-024-10414-z

**Published:** 2025-01-07

**Authors:** Vallery Ogello, Paul Mwangi, Zachary Kwena, Nicholas Thuo, Catherine Makokha, Emmah Owidi, Nelson Muteti, Catherine Kiptinness, Nelly R. Mugo, Kenneth Ngure

**Affiliations:** 1 Center for Clinical Research, Partners in Health Research and Development, Kenya Medical Research Institute, Nairobi, Kenya; 2 Center for Microbiology Research, Kenya Medical Research Institute, Kisumu, Kenya; 3 Kenya Medical Research Institute, Nairobi, Kenya; 4 Department of Global Health, University of Washington, Seattle, USA; 5 School of Public Health, Jomo Kenyatta University of Agriculture and Technology, Nairobi, Kenya

**Keywords:** Long-acting PrEP, Pregnancy and lactation, Preferences, Acceptability, HIV prevention, Kenya, Women users of health services

## Abstract

**Background:**

Increased risk of HIV acquisition during pregnancy and lactation among women is evident, necessitating their inclusion in the evaluation of new HIV prevention interventions. Pregnant and postpartum women specifically face challenges with oral PrEP associated with stigma, and the burden of using other tablets. Long-acting products may address challenges related to oral PrEP, however, there is limited data on product-specific preferences and acceptability among pregnant and lactating women.

**Methods:**

We conducted a mixed-method study to assess the preferences and acceptability of long-acting PrEP modalities either under development or already established among pregnant and lactating women. We conducted quantitative surveys (*n* = 434) and in-depth interviews (*n* = 80) in central and western Kenya. We used descriptive statistics and categorical variables to summarize frequencies and proportions. Inductive and deductive content analytic approaches were used for in-depth interviews.

**Results:**

The median age of respondents was 25 years (IQR 19.3–31.0). Majority were married (263/434, 61%), had completed high school (222/434, 51%), with no condoms use in the prior 3 months (348/434, 80%). The most preferred PrEP formulations were injectable (251/434, 57%) and implantable (175/434, 40%) options. Participants who preferred injectable PrEP had 8.56 times higher odds of considering ease of use as a reason. (aOR = 8.56, 95% CI [3.81–20.48]) and 3.71 odds of choosing perceived discreteness (aOR = 3.71, 95% CI (1.57–9.97)) as their preference reasons. Participants who preferred Implant for HIV prevention had 2.31 odds of considering it due to perceived effectiveness in preventing HIV as a preference reason (aOR = 2.31, 95% CI (1.21—4.66)) and 2.53-fold of considering discreteness as a preference reason (aOR = 2.53, 95% CI (1.46—4.59)). From the in-depth interviews, women reported prospective acceptability due to the perceived convenience of LA products, perceived effectiveness, reduced cost, improved privacy, and reduced stigma. Women had concerns regarding the safety and efficacy of the products during pregnancy and lactation.

**Conclusion:**

Acceptability of LA products underscores the importance of considering the unique needs of pregnant and breastfeeding women in the development of future prevention interventions**.** Aligning preferences and needs would enhance the uptake and adherence outcomes of HIV prevention products.

**Supplementary Information:**

The online version contains supplementary material available at 10.1186/s12879-024-10414-z.

## Introduction

Women and girls (all ages) accounted for 44% of all new HIV infections globally in 2023 [1]. In sub-Saharan Africa, HIV incidence continues to decline among the majority of sub-populations except women [2]. The Adolescent girls and young women (AGYW) in the region carry a disproportional burden of incident HIV infections, and in 2022 accounted for 63% of the 670,000 new infections in the region [3]. The risk of HIV transmission is elevated during pregnancy and post-partum due to biological reasons associated with complex interactions between the immune system and sexual hormones, and studies have shown high HIV incidences during these maternal periods, especially among women with unknown partner status [4–7]. Pregnant and post-partum women in the region also experience social risk factors related to limited decision-making power such as *‘lack of control’* and social-cultural circumstances which often increase their susceptibility to HIV [8]. In the absence of HIV prevention interventions, the rate of vertical transmission ranges from 15 to 45% [9], therefore, HIV prevention interventions are crucial to reducing the risk of HIV acquisition in women and transmissions to infant [10].

Oral pre-exposure prophylaxis with tenofovir/emtricitabine(PrEP TDF/FTC) is effective and safe during pregnancy and lactation [11] and is currently the standard of care for pregnant women at risk for HIV infection [12]. Adherence to daily oral PrEP continues to be a challenge often due to logistical challenges and HIV risk perception [13, 14]. Pregnant and post-partum women specifically face challenges with adherence to oral PrEP associated with disclosure of PrEP use, stigma, and the burden of using other tablets during pregnancy or post-partum [15]. Long-acting products have the potential to address challenges related to oral PrEP such as daily administration and stigma concerns [16]. The effectiveness of oral PrEP depends on daily and consistent use, hence a less user-dependent formulation could bolster adherence [17, 18].

The World Health Organization approves the use of long-acting (LA) HIV prevention tools as an additional prevention choice for people at substantial risk of HIV and several new technologies are currently under research [19, 20]. Case in point, clinical trials have shown long-acting cabotegravir (CAB-LA) to be superior to oral PrEP with TDF/FTC among women as it reduced the rate of HIV incidences [21]. Two clinical trials of the dapivirine vaginal ring (DPV-VR) the Ring and ASPIRE study demonstrated an HIV risk reduction of 35% and 27%, respectively, among women that used the DPV-VR compared to a match placebo ring [22, 23]. Islatravir implant (MK-8591) for annual use has demonstrated safety and is under evaluation for effectiveness [24]. Despite the attentiveness to these biomedical advancements, little is known about the preferences of some categories of potential end-users which is critical in determining the success of an intervention [25]. Various studies have assessed the acceptability and preferences of LA HIV prevention technologies among different populations such as oral PrEP users, adolescent girls and young women, and pregnant and post-partum women where high acceptability and varied preferences are reported [26–29]. However, there exists limited data on product-specific preferences and how individuals within these groups prioritize or choose between different LA PrEP options. We contextualize preference and acceptability and associated reasons within a real-world setting.

This mixed method analysis explores product-specific preferences of LA HIV prevention technologies during pregnancy and lactation framed within the context of the theoretical framework of acceptability(TFA) [30]. TFA was developed to assess components of healthcare interventions at different time points. In this study, we assess end user prospective acceptability (pre-intervention) through the lens of key constructs (affective attitude, perceived effectiveness, opportunity cost, ethicality, self-efficacy, and burden) to understand multiple dimensions of LA HIV prevention products acceptability during pregnancy and lactation. This evidence is essential to design biomedical prevention products that are responsive to pregnant and lactating women's unique HIV prevention needs and life circumstances.

## Methods

### Study setting and population

Between October 2022 and April 2023, we conducted a cross-sectional mixed method study among pregnant and lactating women from the central region, Kiambu, (a peri-urban, moderate HIV prevalence setting) and western regions, Kisumu (a rural, high HIV prevalence setting) Kenya. The two counties have reported HIV prevalence of 4.0% and 16.3% for Kiambu and Kisumu, respectively [31]. Sites in Kiambu included Thika Level 5 Hospital and Kiambu County Hospital, while sites in Kisumu included Kisumu County Hospital and Ahero Sub-County Hospital. The sites have ethnically diverse populations and high volume of women attending antenatal clinics/mother–child health clinics (ANC/MCH) services. Additionally, the facilities offer oral PrEP as part of the maternal health services in the prevention of mother-to-child transmission clinic (PMTCT) as approved by the Ministry of Health. Services provided at these health facilities include routine antenatal check-ups, child welfare clinics, HIV testing, and counseling. Health workers delivering these services depend on the size and resources of the facility which includes nurses, clinical care providers, and HIV testing counselors.

Participants were eligible if they were pregnant or lactating, HIV uninfected, ≥ 15 years of age, willing to provide written informed consent, and accessing ANC/MCH services at the time of enrollment.

### Recruitment and data collection

In the initial data collection phase, we conducted in-depth interviews using semi-structured interview guides informed by the theoretical framework of acceptability to understand prospective product preference and acceptability through the lens of affective attitude, perceived effectiveness, self-efficacy, opportunity cost, ethicality, and burden. We conducted a total of 80 interviews (*n* = 40 in Thika and *n* = 40 in Kisumu) based on the principle of saturation, where additional interviews were stopped once no new data emerged [32]. Participants were recruited for in-depth interviews at respective healthcare facilities during their maternal health visits with the help of the facility staff offering prenatal and postnatal care services. We used purposive sampling to select women who were eligible to participate in the interviews. Interviews were conducted by experienced female researchers in a private space with the participant and the interviewer only. Given the sensitive nature of women's health discussions during their maternal periods, female interviewers ensured participants were comfortable and safe in sharing their preferences and life circumstances. In-depth Interviews were completed in the participants' preferred language either Swahili or English. All interview guides were in English and experienced interviewers simultaneously translated the guides into Swahili among participants who preferred the Swahili language. All interviewers were trained, and conducted role plays and interview guides pre-piloted to ensure consistency in how the interviews were administered. The median interview duration was 34 min [IQR 20–53 min].

During the second data collection phase, we conducted structured quantitative surveys with up to 434 pregnant and lactating women (*n* = 217 in Kisumu and *n* = 217 in Kiambu). Women participating in these structured interviews were different from those who participated in the in-depth interviews for complementary data and to ensure that each phase added unique perspectives to the study. We estimated our sample size based on 71% acceptability of LA antiretrovirals among non-US participants with a 4.5% margin of error and 95% confidence interval [33, 34]. Women were approached while accessing either ANC/MCH services, and surveys were conducted in private spaces at the participant's preferred date and time at the respective facilities. Surveys were completed by experienced research assistants in the participant's preferred language of either English or Swahili. Women were purposively sampled to represent those who are either pregnant (*n* = 217) or lactating (*n* = 217), and varied age stratification e.g. (15-18), (19-24), (25-30) and above 30 years, to ensure diverse opinions. We used an equal proportion of pregnant and lactating women across the two sites to ensure each site contributed equally to the analysis avoiding bias due to unequal weighting. Data was collected in real-time using an open data kit (ODK) [35] using a pre-piloted questionnaire (supplementary material). A trained data research staff reviewed survey data for correctness and completeness. To ensure both the validity and reliability of the survey tools, survey tools were reviewed by statistical analysts to confirm that they accurately measured the intended constructs. Additionally, data survey tools were pilot-tested from various facilities and sites, and data was consistently monitored throughout the data collection.

The integration of qualitative and quantitative data was achieved by identifying concepts that enriched or expanded upon the quantitative results, to provide a more comprehensive understanding of the findings.

#### Long-acting HIV prevention products

We assessed preferences for four long-acting products: injectable PrEP (CAB-LA), the implant (islatravir [MK-8591]), monthly oral PrEP (MK-8527), and weekly oral PrEP. Additionally, the dapivirine vaginal ring was included as an established option. In the quantitative surveys, we asked about the level of product preference for each of the long-acting products and defined responses by *(most preferred, preferred, moderately preferred, and not preferred).* All products were mutually exclusive. To understand the depth of preference, we used qualitative methods to confirm or expound on concepts of LA product preferences.

### Data analysis

Descriptive statistics for quantitative data were used to summarize participant demographics and responses about the preferences for long-acting HIV prevention products during pregnancy and lactation. The outcome measured was the preference for each long-acting product, including the vaginal ring, injectable PrEP (CAB-LA), implant, monthly oral PrEP, and weekly oral PrEP. Preferences were categorized into a binary scale, where "most preferred," "preferred," or "moderately preferred" indicated a preference for the product, and others were categorized as not preferring the product. Categorical variables were summarized using frequencies and proportions, and continuous measures were summarized using means and standard deviations or medians and interquartile ranges, as appropriate. The univariate and multivariate logistic model was used to assess the ease of use, dosage frequency, effective prevention of HIV, discreteness, and accessibility factors associated with the preference of each long-acting HIV prevention method, respectively. We included all these key predictors, along with age, participant category (lactating or pregnant), marital status, income status, and HIV risk perception (e.g., not being worried about acquiring HIV in the past three months) to ensure more accurate identification of key factors associated with the preference for each long-acting HIV prevention product. The logistic regression results were presented using the odds ratio and the corresponding 95% Confidence Interval (CI). Quantitative analyses were conducted using R statistical software (version 4.3.1) (R Foundation for Statistical Computing, Vienna, Austria). The 5% significance level was used for data analysis (*P* ≤ 0.05).

In-depth interviews were analyzed using inductive and deductive content analytic approaches informed by the theoretical framework of acceptability to identify key themes related to the acceptability of LA HIV prevention products. Coding was supported with Dedoose software (version 8.3.35) (sociocultural Research Consultants, LLC, Los Angeles, California, USA.) Five research team members discussed discrepancies of ten transcripts (12%) during the first stage of the coding process until a consensus was reached. Coding disagreements were discussed in a weekly team discussion meeting. After an agreement was reached, all analysts read through all the transcripts and independently coded the interviews using an agreed-upon codebook. Data were categorized into broader themes and sub-themes, condensed, and summarized to extract meaning in the context of preferences and reasons for acceptability during pregnancy and lactation.

### Rigor and trustworthiness

To ensure rigor and trustworthiness, the quality of evidence in this study is generally reflected in the depth of information elicited from individual participants [36]. In addition, after the coding process was complete, we drew matrices to compare our data and explore differences and similarities. To ensure the transferability of our findings we provide a detailed description of our setting, recruitment procedures as well as the context-specific demographic information of study participants.

### Ethical approval

This study involving human participants was reviewed and approved by the Institutional Review Board at Jomo Kenyatta University of Science and Technology (JKUAT-ERC) (JKU/2/4/896B) and was conducted in compliance with ethical principles outlined in the declaration of Helsinki. All participants provided written informed consent. Participants were only identified by unique codes during data collection.

## Results

### Social demographic and behavioral characteristics

Overall, the median age was 25 years (IQR 19.3–31.0 years). The majority were married or cohabiting (263/434, 61%) and had a high school level of education (222/434, 51%). The majority (276/434, 64%) were not employed and reported no condom use in the past 3 months (348/434, 80%). About (153/434, 35%) were worried about acquiring HIV. There were significant differences across the two sites with PrEP use, marital status, and perception of partner trust in HIV prevention (Table 1).

**Table 1:** Socio-demographic and behavioral characteristics of pregnant and breastfeeding women (*p* ≤ 0.05)

	**Kiambu** **(*N* = 217)**	**Kisumu** **(*N* = 217)**	**All** **(*N* = 434)**	***P*-value**
**Age in years**				
15 – 24	102 (47.0%)	101 (46.5%)	203 (46.8%)	0.995
25 – 48	115 (53.0%)	116 (53.5%)	231 (53.2%)	
**Participant category**				
Breastfeeding	107 (49.3%)	109 (50.2%)	216 (49.8%)	0.982
Pregnant	110 (50.7%)	108 (49.8%)	218 (50.2%)	
**Marital status**				
Married/cohabiting	145 (66.8%)	118 (54.4%)	263 (60.6%)	**0.029**
Not married	72 (33.2%)	99 (45.6%)	171 (39.4%)	
**Highest level of education**				
College/University	81 (37.3%)	62 (28.6%)	143 (32.9%)	0.151
Primary	26 (12.0%)	43 (19.8%)	69 (15.9%)	
Secondary	110 (50.7%)	112 (51.6%)	222 (51.2%)	
**Any income**				
No	141 (65.0%)	119 (54.8%)	260 (59.9%)	0.098
Yes	76 (35.0%)	98 (45.2%)	174 (40.1%)	
**Occupation**				
Employed (formal sector)	12 (5.5%)	20 (9.2%)	32 (7.4%)	0.135
Employed (informal sector)	54 (24.9%)	72 (33.2%)	126 (29.0%)	
Not employed	151 (69.6%)	125 (57.6%)	276 (63.6%)	
**Worried of acquiring HIV in the past three months**				
A little worried to worried most of the time	66 (30.4%)	87 (40.1%)	153 (35.3%)	0.108
Not worried	151 (69.6%)	130 (59.9%)	281 (64.7%)	
**Multiple sexual partners**^**a**^				
No	211 (97.2%)	202 (93.1%)	413 (95.2%)	0.132
Yes	6 (2.8%)	15 (6.9%)	21 (4.8%)	
**No condom use in the past three months**				
No	183 (84.3%)	165 (76.0%)	348 (80.2%)	0.095
Rarely to always	34 (15.7%)	52 (24.0%)	86 (19.8%)	
**Confident that their partner will protect them from acquiring HIV**				
No	114 (52.5%)	145 (66.8%)	259 (59.7%)	**0.010**
Yes	103 (47.5%)	72 (33.2%)	175 (40.3%)	
**Ever talked about HIV prevention with their partner**				
No	96 (44.2%)	64 (29.5%)	160 (36.9%)	**0.006**
Yes	121 (55.8%)	153 (70.5%)	274 (63.1%)	
**Ever taken HIV test together with their partner**				
No	94 (43.3%)	69 (31.8%)	163 (37.6%)	**0.046**
Yes	123 (56.7%)	148 (68.2%)	271 (62.4%)	
**Table 1 sociodemographic**				
**Currently using any birth control methods**				
No	176 (81.1%)	160 (73.7%)	336 (77.4%)	0.185
Yes	41 (18.9%)	57 (26.3%)	98 (22.6%)	
**Currently using PrEP**				
No	215 (99.1%)	188 (86.6%)	403 (92.9%)	** < 0.001**
Yes	2 (0.9%)	29 (13.4%)	31 (7.1%)	
**Most influential making decisions concerning your health**				
partner/s or friends or parents or others	82 (37.8%)	77 (35.5%)	159 (36.6%)	0.883
Self	135 (62.2%)	140 (64.5%)	275 (63.4%)	
**HIV Status Primary Sexual Partner**				
No	216 (99.5%)	215 (99.1%)	431 (99.3%)	0.846
Yes	1 (0.5%)	2 (0.9%)	3 (0.7%)	

^a^Having another sexual partner/s other than their primary sexual partner

### Preferences for long-acting HIV prevention products

Overall, injectable and implantable PrEP were the most preferred forms of PrEP across both sites with (251/434, 57%) and (175/434, 40%,) respectively. More participants in Kisumu preferred injectable PrEP (145/434, 67%) compared to Kiambu (106/434, 49%). Most women in Kiambu preferred the monthly oral tablet (83/434, 38%) while Kisumu participants preferred the weekly oral tablet (41/434, 19%). The vaginal ring had the least preferences across both sites (21/434, 4.8%). Preferences for the injectable, monthly, and weekly oral tablets significantly varied across both sites (Table 2).

**Table 2:** Preferences for Long-Acting formulation for HIV prevention (*p* ≤ 0.05)

	**Kiambu** **(*N* = 217)**	**Kisumu** **(*N* = 217)**	**Overall** **(*N* = 434)**	***P*-value**
**Vaginal ring**				
Moderately preferred	30 (13.8%)	44 (20.3%)	74 (17.1%)	0.090
Most preferred	16 (7.4%)	5 (2.3%)	21 (4.8%)	
Not preferred	151 (69.6%)	151 (69.6%)	302 (69.6%)	
Preferred	20 (9.2%)	11 (5.1%)	31 (7.1%)	
**Injectable PrEP**				
Moderately preferred	32 (14.7%)	22 (10.1%)	54 (12.4%)	0.016*
Table 2: preferences of LA products				
Most preferred	106 (48.8%)	145 (66.8%)	251 (57.8%)	
Not preferred	22 (10.1%)	17 (7.8%)	39 (9.0%)	
Preferred	57 (26.3%)	32 (14.7%)	89 (20.5%)	
**Implant**				
Moderately preferred	52 (24.0%)	27 (12.4%)	79 (18.2%)	0.063
Most preferred	75 (34.6%)	100 (46.1%)	175 (40.3%)	
Not preferred	49 (22.6%)	43 (19.8%)	92 (21.2%)	
Preferred	41 (18.9%)	44 (20.3%)	85 (19.6%)	
**Monthly oral pill**				
Moderately preferred	45 (20.7%)	58 (26.7%)	103 (23.7%)	0.004*
Most preferred	83 (38.2%)	64 (29.5%)	147 (33.9%)	
Not preferred	24 (11.1%)	52 (24.0%)	76 (17.5%)	
Preferred	65 (30.0%)	42 (19.4%)	107 (24.7%)	
**Weekly oral pill**				
Moderately preferred	60 (27.6%)	73 (33.6%)	133 (30.6%)	0.002*
Most preferred	17 (7.8%)	41 (18.9%)	58 (13.4%)	
Not preferred	101 (46.5%)	61 (28.1%)	162 (37.3%)	
Preferred	39 (18.0%)	42 (19.4%)	81 (18.7%)	

^*^Level of significance (*P* =  < 0.05)

### Reasons for product preference

Most respondents preferred the products due to perceived ease of use (278/434, 64%) and the dosing frequency (242/434, 56%). The majority did not consider accessibility (416/434, 96%). Perceived ease of use and perceived product effectiveness as reasons for product choice were statistically significant across the two sites (*p* ≤ 0.001). There was no statistical significance across pregnant and lactating women with reasons for preferring a LA method (Table 3).

**Table 3:** Reasons for the most preferred method

		**Regions**			**Population type**		
	**All** **(*N* = 434)**	**Kiambu** **(*N* = 217)**	**Kisumu** **(*N* = 217)**	***p* value**	**Lactating** **(*N* = 216)**	**Pregnant** **(*N* = 218)**	***p* value**
**Ease to use**	278 (64.1%)	178 (82.0%)	100 (46.1%)	**< 0.001**	141 (65.3%)	137 (62.8%)	0.870
**Dosage frequency**	242 (55.8%)	124 (57.1%)	118 (54.4%)	0.845	116 (53.7%)	126 (57.8%)	0.692
**Effective preventing HIV**	119 (27.4%)	35 (16.1%)	84 (38.7%)	**< 0.001**	59 (27.3%)	60 (27.5%)	0.999
**Discreteness privacy**	157 (36.2%)	73 (33.6%)	84 (38.7%)	0.547	77 (35.6%)	80 (36.7%)	0.974
**Accessibility**	18 (4.1%)	11 (5.1%)	7 (3.2%)	0.629	9 (4.2%)	9 (4.1%)	1.000

### Factors associated with preference for long-acting HIV prevention products

Table 4 shows the multivariable logistic model on factors associated with women's preference for long-acting HIV prevention products. Participants who preferred Implant for HIV prevention had 2.31 odds of considering perceived effectiveness in preventing HIV as a preference reason compared to those who did not perceive its effectiveness in preventing HIV (aOR = 2.31, 95% CI (1.21—4.66)) and 2.51 odds of considering discreteness as a preference reason compared to those who did not consider discreteness (aOR = 2.53, 95% CI (1.46—4.59)). Participants who preferred the injectable had higher odds 8.56 of considering it due to ease of use (aOR = 8.56, 95% CI (3.81–20.48)) compared to those who did not consider ease of use and 3.71 odds of choosing perceived discreteness (aOR = 3.71, 95% CI (1.57–9.97)) as their preference reasons compared to those who did perceive discreteness as a reason for its preference. The odds of women preferring the monthly oral pill increased with its dosage frequency compared to those who did not prefer it due to dosage frequency (aOR = 2.03, 95% CI (1.19–3.50)) and perceived effectiveness in HIV prevention compared to those who did not perceive it effective in HIV prevention. (aOR = 4.79, 95% CI (2.19–11.80)). Participants who preferred the weekly oral pill were 0.38 times less likely to report ease of use as a reason for their preference compared to those who did consider it easy to use (aOR = 0.62, 95% CI [0.38–0.99]). Similarly, participants who preferred the vaginal ring were 0.42 times less likely to report dosage frequency as a reason for their preference (aOR = 0.58, 95% CI [0.37–0.89]) (Table 4).

**Table 4:** The multivariable logistic model indicating factors associated with preference for long-acting HIV prevention products

	**Implant**		**Injectable**		**Monthly oral pill**		**Vaginal ring**		**Weekly oral pill**	
Dependent variables	aOR (95% CI)	*p*-value	aOR (95% CI)	*p*-value	aOR (95% CI)	*p*-value	aOR (95% CI)	*p*-value	aOR (95% CI)	*p*-value
Age in years	1.02 (0.97–1.06)	0.470	1.00 (0.93–1.06)	0.883	0.98 (0.94–1.03)	0.426	1.04 (1.00–1.08)	0.054	1.00 (0.97–1.04)	0.884
Participant category										
Breastfeeding	1.00		1.00		1.00		1.00		1.00	
Pregnant	1.40 (0.87–2.26)	0.173	1.46 (0.71–3.07)	0.304	0.94 (0.56–1.58)	0.814	1.09 (0.71–1.68)	0.681	0.97 (0.64–1.47)	0.885
Marital status										
Married/cohabiting	1.00		1.00		1.00		1.00		1.00	
Not married	0.70 (0.41–1.21)	0.204	3.27 (1.31–9.00)	0.015	0.74 (0.41–1.35)	0.327	1.10 (0.67–1.80)	0.716	1.05 (0.65–1.69)	0.841
Highest level of education										
College/University	1.00		1.00		1.00		1.00		1.00	
Primary	0.73 (0.36–1.50)	0.389	1.80 (0.64–5.72)	0.284	1.23 (0.58–2.74)	0.598	0.84 (0.43–1.61)	0.606	2.02 (1.06–3.99)	0.037
Secondary	0.86 (0.48–1.53)	0.608	1.50 (0.65–3.48)	0.339	1.06 (0.58–1.93)	0.850	1.15 (0.70–1.90)	0.594	0.91 (0.56–1.47)	0.695
Any income										
No	1.00		1.00		1.00		1.00		1.00	
Yes	1.09 (0.63–1.87)	0.762	1.88 (0.86–4.28, *p* = 0.123)		1.12 (0.63–2.01)	0.697	0.85 (0.52–1.37)	0.500	1.22 (0.77–1.94)	0.397
Not worried of acquiring HIV in the past three months	1.19 (0.72–1.94)	0.496	0.66 (0.28–1.43)	0.307	1.36 (0.80–2.30)	0.256	0.80 (0.52–1.25)	0.330	1.15 (0.75–1.77)	0.522
Ease to use										
No	1.00		1.00		1.00		1.00		1.00	
Yes	1.38 (0.79–2.40)	0.258	8.56 (3.81–20.48)	< 0.001	1.72 (0.94–3.14)	0.078	0.93 (0.57–1.54)	0.780	0.62 (0.38–0.99)	0.048
Dosage frequency										
No										
Yes	1.14 (0.69–1.87)	0.601	1.92 (0.89–4.23)	0.098	2.03 (1.19–3.50)	0.010	0.58 (0.37–0.89)	0.014	1.29 (0.85–1.96)	0.239
Effective preventing HIV										
No	1.00		1.00		1.00		1.00		1.00	
Yes	2.31 (1.21–4.66)	0.014	2.42 (0.97–6.60)	0.068	4.79 (2.19–11.80)	< 0.001	0.98 (0.57–1.66)	0.946	2.22 (1.31–3.87)	0.004
Discreteness privacy										
No	1.00		1.00		1.00		1.00		1.00	
Yes	2.53 (1.46–4.59)	0.001	3.71 (1.57–9.97)	0.005	1.60 (0.90–2.91)	0.116	0.72 (0.45–1.14)	0.163	1.70 (1.09–2.69)	0.020
Accessibility										
No	1.00		-		-		1.00		1.00	
Yes	1.89 (0.50–12.38)	0.415	-	-	-	-	0.67 (0.18–1.97)	0.499	0.97 (0.35–2.96)	0.961

The model was adjusted for age in years, group (pregnant vs lactating), marital status, income, highest level of education, and worried of acquiring HIV in the past three months

*OR* represents Odds Ratio*, aOR* represents adjusted odds ratio

### Qualitative findings

#### Social demographic characteristics of participants in the in-depth interviews

Of the (*n* = 80) pregnant and breastfeeding women who participated in the in-depth interviews, (54%) were breastfeeding. Participants had a median age was 24 years (IQR 19.8–30.0). The majority (64%) were married and had a median school completion of 12 years (IQR 10.0–12.3) equivalent to high school completion. The majority (43%) had more than 3 children (Table 5).

**Table 5:** Demographic characteristics of participants in the qualitative study

	**Kisumu** **(*N* = 40)**	**Thika** **(*N* = 40)**	**Overall** **(*N* = 80)**
**Age**			
Mean (SD)	25.6 (7.22)	24.3 (6.52)	25.0 (6.87)
Median [IQR]	25.5 [19.8,30.5]	23.0 [19.5,29.3]	24.0 [19.8,30.0]
**Marital status**			
Married	27 (67%)	24 (60%)	51 (64%)
Single	13 (33%)	16 (40%)	29 (36%)
**Level of education(years)**			
Mean (SD)	11.3 (2.87)	11.6 (2.25)	11.5 (2.57)
Median [IQR]	11.0 [10.0,12.0]	12.0 [10.0,13.0]	12.0 [10.0,12.3]
**Pregnant/ breastfeeding**			
Breastfeeding	23 (57%)	20 (50%)	43 (54%)
Pregnant	17 (43%)	20 (50%)	37 (46%)
**Parity**			
None	9 (22%)	16 (40%)	25 (31%)
1–2	11 (28%)	10 (25%)	21 (26%)
≥ 3	20 (50%)	14 (35%)	34 (43%)

### Qualitative summary

Overall injectable and implant formulations were the most preferred across both sites by about (*n* = 36/80) and (*n* = 24/80) respectively. The monthly tablets were preferred by (*n* = 17/80). The vaginal ring had the least preferences (*n* = 3/80). Acceptability and preference of injectable PrEP were due to less worry about daily pill taking or forgetfulness, discreteness, and perceived high-level efficacy. Preference for implants was due to the longer duration that would reduce hospital visits and lower the burden of travel costs. Participants who preferred monthly oral pills reported perceived minimal side effects and ease of discontinuation during seasons of reduced HIV risk. Few who preferred the vaginal ring was because it was perceived to be painless. Despite the high acceptability, women expressed perceived concerns about the use of LA HIV prevention products associated with contraception products of similar modalities, and concerns on safety and efficacy during pregnancy and lactation. We describe these results in detail using TFA constructs, affective attitude, perceived effectiveness, opportunity cost, user experience, ethical consequences, self-efficacy, and burden.

### Affective attitude

#### Convenience of long-acting HIV prevention products

Overall, women were willing to use varied HIV prevention products that were convenient to suit their needs and circumstances. Injectables and implants were the most preferred HIV prevention products because they were perceived as easy to use due to the perceived absence of pill burden and less worry of taking medication, which would contribute to perceived improved adherence. Women felt confident that these formulations would give them ‘peace of mind’ and less worry about acquiring HIV even with a partner of unknown HIV status. Among these women, the monthly pill was perceived to have an adherence challenge due to forgetfulness. A participant in Kisumu who was using daily oral PrEP echoed that she would gladly switch to injectable due to forgetfulness of the daily medication.

*“When we have the injection, I will have to stop using the current pills [daily oral PrEP] that I am using since there is a possibility of forgetting especially when I travel” [20-year-old, lactating, Kisumu].*

*“No one trusts their partner, especially us women when pregnant or breastfeeding, especially in the early months, after you have delivered your baby, the first three months, most of the times, men go out (cheat)… we will feel protected” [24-year-old, pregnant, Thika]*

Among 17 women*,* the monthly pill was perceived to be easy to use as it would allow preventive effective adherence to PrEP, and they would be able to stop whenever they felt they had reduced HIV risk. A monthly pill was also perceived to have minimal side effects and in case of any notable side effects, they would easily stop. In addition, the preference for monthly oral tablets was also due to fear of needle prick with the use of Injectables.

*“Once you use that pill, let’s say it is monthly, within that month it gives you time if it is short duration as compared to that one year, so it will help you even to change to another one if there are the side effects, yeah.” [29-year-old, lactating, Kisumu]*

The vaginal ring had overall low acceptability, few (*n* = 3) who preferred it cited it being painless compared to injectables and implants that are inserted under the skin. Vaginal ring was also perceived to have minimal side effects and reduce pill burden.

*“The only one I think I will prefer is the ring because for tablets I am afraid of it. The injection also, the pain…but for the ring, it is something that you insert it, and you are done, I don’t think it can stress you so much”. [21-year-old, lactating, Thika]*

#### Enhanced HIV prevention choice

The long-acting HIV prevention products were also acceptable as women felt they would have the ' power *of choice’*. For instance, a woman highlighted that oral PrEP was the only available HIV prevention product at the health facilities. Women were particularly happy about the availability of varied products that would allow them to pick what they wanted based on the formulation or dosing frequencies.

*“If it comes in different forms, I will be the person to choose the one I want to use, so yes it will help a lot of people. But this one, the daily pill it's already decided, you are going to take it daily. So, what if they don't want to use the daily pill, they can still choose another.” [28-year-old, pregnant, Kisumu]*

### Perceived effectiveness

#### Perceived high-level efficacy

The use of long-acting HIV prevention products; specifically, the injectable was perceived as highly efficacious compared to other methods, citing *‘injection has more power than the tablet ever’*. Women highlighted that a drug that is infused into the body goes directly into the bloodstream and therefore had a high efficacy compared to other localized formulations such as the ring.

*“I think it won’t be that effective like the injection one, I feel maybe it will just be there, I’ll be like is it really working? because for the injection one, I know it is injected and it is going in the blood, but for the ring I’ll be suspicious of myself”. [25-year-old, lactating, Kisumu]*

#### Varied levels of perceived HIV risk during pregnancy and lactation

Women highlighted the different HIV risks during periods of pregnancy and lactation. Lactating women were perceived to have a higher likelihood of infecting their children during lactation without their knowledge compared to pregnant women. For this reason, lactating women were perceived to have a higher preference for a longer formulation such as injectable/implant. Pregnant women on the other hand were reported to have limited preference for oral pills due to the burden of using other tablets during pregnancy.

*“During breastfeeding, I can use injection, let us say the injection is available I can use it because when I am breastfeeding, I will be taking the baby to the clinic every month and get the injection so after I have stopped breastfeeding then I can use the implant”. [32-year-old, lactating, Kisumu]*

Further, women described their high HIV risk associated with having different sexual partners, partner having other partners, and the risk of infecting their babies as the reasons why the HIV prevention products were acceptable to them.

*“I’m married, and my husband has many sexual partners, and I also have different sexual partners so you see here I must use it because I don’t want to get infected… I’m not done with having babies and I will still want to use it when I’m pregnant so that I don’t infect the baby and while breastfeeding, I should not infect the baby.” [28-year-old, pregnant, Kisumu]*

### Opportunity cost

#### Time-saving and reduced cost

The implant was highly perceived as a facilitator of opportunity cost. First, it was perceived to be of a longer duration that would reduce hospital visits, which would enable them to engage in other tasks. In addition, reduced hospital visits would help reduce the burden of travel costs and ultimately save time and money. Lactating women preferred implants also because of the burden of breastfeeding and childcare after delivery. Women felt that the implant would give them ample time to take care of their children during these seasons.

*“I think it will be good; you will not have to keep returning to the hospital maybe if you come this one month, you know the next time you will return will be after a few months. Then maybe if you are somewhere far… where you cannot access such services… If the long-term product will last me a few months, I know by the time I return and get another injection or implant, I will feel safe and secure [24-year-old, pregnant, Thika].*

Regarding the perception that implant was of a longer duration, women mentioned that they would not need to worry about their jobs or getting permission from their workplace often because it would take them a longer time before the next visit. Women particularly highlighted the challenges of routine hospital visits.

*“You know when you prevent yourself for long. If it is short-term, let’s say you are working, you don’t have much time to go back to the hospital. But the long term is better because you know before the time stipulated ends, you will have planned for the time to return.” [25-year-old, pregnant, Thika]*

### Experience and satisfaction

#### Experiences with contraception methods of similar modality

Women also associated the contraceptive methods that they were using for pregnancy prevention and compared them to the long-acting HIV prevention products. For instance, a client using the implant for pregnancy prevention preferred the implant (Islatavir) for HIV prevention because of her good experience with the implant contraception citing *“I have never seen an implant[contraception] fail, so even the implant PrEP cannot fail’.*

In addition, women compared formulations of the same modality that is (CAB-LA to Depo provera, Islatavir to Implanon, daily progestin oral contraceptives to monthly pills, and vaginal ring to intrauterine device (IUD). This comparison was largely attributed to the experiences women had while using pregnancy contraceptive methods.

*“Basically, it is because I have experience in using the implant family planning for duration of three years; it has no side effects on my body and also it is a simple procedure. It is not a procedure that will make me feel uncomfortable because it is on the arm. For tablets, I can easily forget, vaginal ring is not for me” [ 34-year-old, lactating, Thika]*

Further, the similarity of the modalities shaped the perception of the product's perceived efficacy. Women who got pregnant while on a contraceptive of the same modality perceived long-acting products of a similar modality to have low-level efficacy in HIV prevention. In essence, negative experiences with these contraception methods contributed to negative perceptions about the efficacy of the HIV prevention products.

*“It was in the media, especially radio. I gave birth to triplets and one of them was holding the coil [IUD] firmly in his hands. So, I feel taking the pills is easy even my preferred family planning method are the pills. So according to me, I feel that” taking the PrEP pills is very easy. I may think that it is okay to take PrEP injectable and maybe my blood is light [potential of being resistant] and that is how I will get the virus”. [35-year-old, lactating, Kisumu]*

### Ethical consequences

#### Risk of using HIV prevention methods during pregnancy

Pregnant women reported concerns about medication use during pregnancy. First, women reported that pregnant women are ‘not allowed’ to take any form of medication without prescription and there may be concerns about accepting to use LA products. Women further described the risks of medication use, such as causing pregnancy loss or affecting the baby.

*“Because sometimes the doctors usually tell us not to use medicines when we are pregnant, medicines are not good. [23-year-old, pregnant, Thika]*

#### Improved privacy and confidentiality

Women also felt that LA HIV prevention products support privacy and confidentiality. Especially for the implant and injectables. Clients felt that using these methods would protect them from unintended disclosure of PrEP use and stigma associated with HIV prevention, especially from their partners and peers.

*“It will be discreet, because it is something I have on my body , it will not be easy for him to know but the ring he will feel something ,for the pills , I will have them in the house…and he can hide it so on the day you are to use it ,it will not be there”.[30-year-old, pregnant, Kisumu]*

Women emphasized privacy as important due to the existing stigma associated with HIV prevention. Women reported that the use of daily oral PrEP is often confused with ART use, hence the use of LA HIV prevention products would be discreet and would protect them from potential stigma. In addition, women felt that they had control and could use the product even when their partners were not aware because of discreetness.

*“I choose injection because I just like it, you know for injection once given I just leave, but I fear oral medication that is why I choose injection. Again, when you use injection no one will know you are using it, but sometimes you can use medication[pills] and then someone finds you taking it. This person will wonder why you are taking medication[pills]…. If you had a husband he would not find out, that is why I like injections more than the medication[pills] .[27-year-old, lactating, Kisumu]*

### Self-efficacy

#### Ease of use of long-acting HIV prevention products

Women perceived the use of long-acting products as easy to use while giving varied reasons based on their acceptability. Implants and injectables were perceived as easy to use because they are administered by a trained health provider, and the administration would take a shorter time. In addition, the implant was perceived to be removable whenever there were any experienced side effects.

*“The process of it being infused on the arm is so easy, it is not painful then you find that the doctors or nurses assigned can put it so easily and can be removed at any time. If you feel that it is affecting you, then you can go and be removed any time, but injections cannot be removed” [26-year-old, lactating, Kisumu]*

Injectables were also perceived to be easy to use due to the reduced burden of taking medication. Ease of use was also associated with the ability to align LA PrEP visits to clinic visits. Implying that the 2–3 months dosing frequency would work well with their antenatal visits.

The monthly pill was also perceived to be easy to use because they are only taken once in a month compared to the daily oral pill, hence it would be easy for them to remember to take.

*“For most pregnant women, they don’t like taking medication, like for me, I have a prescription for calcium supplements to take for a whole month, they are really stressing me because I must wake up daily, eat first then take them. So, I think they will feel uncomfortable, then again morning sickness, vomiting, once you take that medication you will vomit. “Will it have helped you… No!” [16-year-old, pregnant, Thika]*

Low self-efficacy of the vaginal ring was attributed to the lack of knowledge about its use and how it is inserted. Women felt that it may be difficult to learn how to use the vaginal ring given they had little information about it. Some also reported fears of vaginal ring insertion if done by a provider due to privacy concerns.

*“The one that I dislike is the vaginal ring, it is the first time to hear about it, but I do not know how to use it, maybe I may prefer to use it in future if I would know how it is used” [16-year-old, pregnant, Kisumu]*

### Burden

#### Concerns about product efficacy and safety

Safety and efficacy were overall key concerns among women as they cited limited knowledge about the products. Among pregnant women, the main concerns were about effect on the unborn child such as birth defects, bleeding or miscarriage, and preterm birth. Among lactating women, the main concern was whether the long-acting HIV prevention products would be excreted in breastmilk and whether that would have any effect on the breastfeeding child. In addition, women highlighted that they would use these products if they were provided with evidence of safety and efficacy during pregnancy and breastfeeding.

*“When breastfeeding the medication should not be produced with the breastmilk so that it does not affect the baby because she is young, innocent, and someone that does not know anything. So, it can affect them and maybe your baby may die or get sick because of using it” [18-year-old, lactating, Thika]*

#### Concerns about product use

Some women were concerned about injectables being irreversible highlighting that in case of any side effects, it could not be removed. Among such women, non-systemic products such as the monthly oral pill, and the vaginal ring were preferred due to the perceived ability to be removed/stopped if side effects are experienced. For the implant, a few clients reported fear of minor surgery in the arm due to cosmetic reasons.

*“The one I dislike the most amongst all these are the injections. The rest I can try except injections. No! Because when it is done it is done, there is no going back but the ring is good you can go back. The monthly oral tablet you can stop for the next months and then the next month you can start taking the oral pill or look for another method but injections when you have been injected it is just like* that” *[26-year-old, lactating, Kisumu]*

For the vaginal ring, women were mostly worried about how it is inserted and the misconception that it required skills since its effectiveness would depend on how it is inserted highlighting the potential for seroconversion with poor insertion, or ‘disappearance’. Several women reported fears about the ring not being discrete and the potential of it being felt by the sexual partner. Further women thought that the ring could move during sex, or that it could cause vaginal infections or other infections such as cervical cancer. Additionally, some pregnant women felt the ring may not be safe during pregnancy due to some delicate pregnancies and the potential of it interfering during childbirth.

*“The vaginal one, for someone who has so many sexual partners like me, and first the fear of the vaginal ring disappearing in the vagina, if it goes deep into the vagina and disappears and what if it can bring infection, I don’t know like I’m having unprotected sex with someone who had an infection and where the ring is placed can make me have the same infection he had” (28-year-old, pregnant, Kisumu)*

Participant perspectives describe high interest in LA HIV prevention products that would fit their HIV prevention needs and life circumstances during pregnancy and breastfeeding.

## Discussion

We used mixed-method analysis to explicate preferences and acceptability of long-acting HIV prevention products framed within the context of TFA in two sites in Kenya. Injectable PrEP was the most preferred followed by implants, while the vaginal ring had the least preference. The finding was also consistent with our qualitative findings. Reasons for preferring the LA products were largely associated with ease of use, dosing frequency, and perceived efficacy in HIV prevention. From our in-depth interviews, women perceived LA products as acceptable to them due to reduced worry about HIV acquisition, privacy, and reduced stigma. However, women were concerned about the safety and efficacy of LA products which were associated with the experiences of existing contraception methods. This evidence highlights the potential of these products' use among this population.

The high preference for injectables and implants in this study is similar to other studies conducted in South Africa, Eswatini, and Kenya among a similar population of pregnant and lactating women on oral PrEP and adolescent girls and young women who have shown a higher preference for longer-acting formulations [26, 29]. Our paper contributes to the growing body of knowledge with perspectives within a real-world setting. High preference for injectables was mostly associated with perceived ease of use, effectiveness at preventing HIV, and a longer duration. This was further explained in our qualitative findings where women felt that products that are infused in the body had *‘high-level efficacy’* compared to other products such as the ring and the pills. Essentially, injectable PrEP(CAB-LA) has been shown to be safe, efficacious, and superior to oral PrEP, especially among cis-gender women, men who have sex with men, and transgender women [21, 37, 38]. However, there remains a critical gap among pregnant and breastfeeding women who have been historically underrepresented in clinical trials which poses delays in understanding safety and efficacy of injectable PrEP among them [39]. Given this evidence, future clinical trials on injectable PrEP may consider efficacy among pregnant and lactating women in similar settings.

Women in Kisumu had a higher preference for injectables compared to Kiambu. This may be linked to the high oral PrEP use reported in Kisumu compared to Kiambu suggesting that women who are more familiar with oral PrEP perceived longer-acting method as easy to use. In addition, Kisumu exhibits high HIV prevalence compared to Kiambu [31]. Hence, within high burden HIV settings injectable and oral PrEP may be highly preferred. The high acceptability of LA products was also due to time-saving and reduced cost. For instance, the implant was seen as an enabler of opportunity costs because of its perceived longer duration, which would reduce hospital visits and save time and money, allowing them to continue with economic activities. Women in Africa have reported adherence challenges to oral PrEP related to dosing frequency and logistical concerns [40]. Our study highlights the potential of LA products addressing these challenges to ultimately benefit women.

Studies have shown increased HIV incidence during pregnancy and breastfeeding [41] and the need to explore risk-related factors when choosing a HIV prevention method for this population [42]. In our setting, women were cognizant of their social circumstances that put them at risk during these periods which are similar to those reported in a qualitative study in sub-Saharan Africa [43]. Uniquely, in our setting some women also reported their sexual factors that increased their HIV risk. Even so, women have previously struggled with HIV risk perception due to miscalculations of risk and a lack of knowledge [44]. Women also reported important ethical consequences related to medication use during pregnancy and breastfeeding based on the perception of not using medication as always advised by healthcare providers. Comparatively, a qualitative study among similar population in other sub-Saharan African countries reported ethical consequences related to cultural and social norms [45]. These ethical issues have the potential to influence the use of new biomedical HIV prevention methods as has been the case for oral PrEP [46]. Future implementation of LA products may incorporate strategies that address ethical concerns.

Overall, the vaginal ring had low acceptability. Similarly, a systematic review showed overall low hypothetical acceptability of the ring with high acceptability among vaginal ring users [47]. In our study, women perceived the ring as difficult to use since it required vaginal insertion and would potentially put them at risk of vaginal infections or intimate partner violence. Of note, is that women in our study had not seen or experienced the ring, and lack of information on its use could have influenced the low acceptability. A qualitative study conducted within a phase III trial conducted in 4 African countries including South Africa, Malawi, Uganda, and Zimbabwe found high acceptability of the ring after its use with initial fears similar to those reported in our study [48]. Interestingly, in that study, women grew to like the ring after its use. Hence prospective and retrospective acceptability may differ. Further, women were also concerned about the safety of the LA products citing fears of *‘miscarriage’ or ‘birth defects’* among pregnant women and effects on breastfeeding children among lactating women. Understanding the safety profile of these LA products is essential to promote use among this populace. In addition, fears of side effects were largely associated with the familiarity of contraception use of similar modalities. Future rollout of LA HIV prevention products should consider education and sensitization to dispel misinformation regarding existing product modalities.

This study had important strengths and limitations. Principally, this study was conducted in two different geographical regions in Kenya and four different ANC/MCH clinics which may increase the generalizability of our findings. In addition, the use of mixed methods allowed us to explain the depth of preferences in our quantitative findings. The primary limitation of this study is that we only report prospective acceptability which may be different in a real-world scenario with actual product use. In addition, the participant selection was biased towards individuals actively seeking care at the time of recruitment, which may result in differing perspectives compared to those not currently engaged in care. Lastly, our study only shows acceptability among pregnant and lactating women that may be different among other priority populations of PrEP users. Despite the limitations, our study establishes an important baseline understanding of acceptability and preferences, which is essential in evaluating future interventions and the effectiveness of long-acting PrEP uptake among pregnant and lactating women.

## Conclusions

Our mixed methods study explains in depth the reasons for preferences and acceptability of LA HIV prevention products framed in the context of TFA. We put into perspective the voices of pregnant and breastfeeding women in a real-world setting to help guide researchers and implementors in their efforts to create products that are responsive and acceptable among end users. Understanding the preference and acceptability of LA products provides crucial evidence for designing effective interventions while recognizing unique needs among pregnant and breastfeeding women and their life circumstances. For this critical populace to benefit from new biomedical HIV prevention technologies, aligning preferences and needs would enhance the uptake and adherence outcomes of HIV prevention products.

## Supplementary Information

Supplementary Material 1.

## Data Availability

Deidentified data will be available upon author request and under appropriate data-sharing agreements to those who provide a methodologically sound proposal.
