# Supplementary material for: Preferences for and acceptability of long-acting HIV prevention products among pregnant and lactating women accessing health services in Kenya: a mixed method cross-sectional analysis
Source: BMC Infect Dis. 2025 Jan 7;25:37. doi: 10.1186/s12879-024-10414-z (PMC11705746; doi:10.1186/s12879-024-10414-z)
Supplement: Supplementary file 1 — Supplementary Material 1. [file 12879_2024_10414_MOESM1_ESM.docx]

**Stakeholder Considerations for Research and Implementation of Long-acting HIV Prevention Products during Pregnancy and Lactation in Kenya**

**Enrollment**

1. Date enrolment consent signed: dd/mm/yy

Participant ID:

1. Name of facility

- Kiambu hospital
- Thika Level 5 hospital
- Kisumu County hospital
- Ahero subcounty hospital

**DEMOGRAPHIC CHARACTERISTICS**

1. Date of birth: DD/MM/YY

Date of Birth OR age in completed years

1. Current marital status

- Single never married
- Married (monogamous) or cohabiting
- Married (polygamous) or cohabiting
- separated
- divorced
- widowed
- Other, specify: _________

1. Are you currently living with her husband/primary partner?

- yes
- no
- N/A, no partner

1. Are you currently pregnant?

- Yes
- No
- If currently pregnant skip 5 and 7

4a. If yes, what is the gestation of the pregnancy(months)

1. Are you currently breast feeding?

- yes
- no

5a. If yes, what is the age of the child(months)

1. How many biological children do you have (count total number of living children)

6a. Are you interested in having (more) children?

- yes
- no
- don’t know

1. Are you currently using any of the following birth control methods

- Injectable
- Oral contraceptive pill
- IUD
- Tubal litigation
- Emergency contraceptive pills
- Condoms
- Traditional/rhythm method
- Diaphragm/gel
- None

1. Does your husband/primary partner provide you with financial and/or material support?

- yes
- no
- N/A, no partner

1. What is your highest level of education you have attended?

- Primary school not completed
- Completed primary school
- Secondary school not complete
- Secondary school complete
- Attended college or university

1. Do you own a mobile phone?

- Yes, her own phone
- Yes, but it is shared with other people
- No

1. Do you earn an income of your own?

- yes
- no

1. Approximately how much money do you make in a month?

- 0 (no income of her own)
- Ksh 1-5000
- Ksh 5001-10000
- Ksh >10000

12a. What is your job/occupation? ***(Choose primary employment if more than one option applies)***

- Student
- Employment with a steady salary/formal sector (e.g., teaching, office work)
- Employment without a steady salary/informal sector (e.g., trader, selling goods)
- Parent caring for own child//housewife
- Household help/childcare for others
- Unemployed/no job
- Other, specify: _______________

1. Have you ever taken a medication daily for more than one week?

- yes
- no

13 a. Which medication was it?

- Contraceptives
- Antibiotics
- PrEP
- Other supplements
- Other, specify

**HIV Risk perception**

1. In the past three months have you been worried of acquiring HIV?

- Not worried
- A little worried
- Sometimes worried
- Worried most of the time

1. Do you have other sexual partners apart from your primary sexual partner?

- yes
- No
- No primary sexual partner

1. In the past three months what made you think that you are at risk of acquiring HIV?

- Having a new partner
- Alcohol use
- Partner has other partners
- Not using condoms
- Suspect my partner of having HIV
- Nothing
- Other, specify

1. The following questions are about how you feel about HIV/AIDS. Please tell us if you strongly agree, agree, disagree, or strongly disagree on the following questions
   1. I have a feeling that I can get HIV

- Strongly agree
- Agree
- Neutral
- Disagree
- Strongly disagree
  1. It is possible that I am at high risk of getting HIV than my friends
- Strongly agree
- Agree
- Neutral
- Disagree
- Strongly disagree
  1. My sexual behaviors put me at risk of acquiring HIV
- Strongly agree
- Agree
- Neutral
- Disagree
- Strongly disagree
  1. I can protect myself against HIV
- Strongly agree
- Agree
- Neutral
- Disagree
- Strongly disagree
  1. My partners behavior puts me at risk of acquiring HIV
- Strongly agree
- Agree
- Neutral
- Disagree
- Strongly disagree
  1. I am confident that my partner will protect me from acquiring HIV
- Strongly agree
- Agree
- Neutral
- Disagree
- Strongly disagree
  1. If I use PrEP, I will be less worried of acquiring HIV?

- Strongly agree
- Agree
- Disagree
- Strongly

**Social Context**

1. We would like to know more about your relationship with your sexual partner.
   1. How old is your partner
   2. Have you ever talked about HIV prevention with your partner

- yes
- no
  1. Have you and your partner taken HIV test together
- yes
- no
  1. What is the HIV status of your primary sexual partner?

- He is HIV negative
- He is living with HIV
- I don’t know
- He is not aware

***Skip if partner not living with HIV*** If living with HIV. Is he currently taking ART?

- yes
- no

1. In the past three months have you had sex with any other partner apart from your primary sexual partner?

- Yes
- No

1. Some people have a challenge using condoms, in the past three months how can you describe your condom use?

- I have not used condoms
- Rarely used condoms
- Sometimes used condoms
- Always used condoms

1. In the past three months have you had sex with your partner because of material or financial support?

- yes
- no

1. In the next set of questions please tell me if you strongly agree, agree, disagree, or strongly disagree
   1. If I tell my partner to use condoms, he will get angry

- Strongly agree
- Agree
- Disagree
- Strongly disagree
- Don’t know
  1. Most of the time we do what my partner wants
- Strongly agree
- Agree
- Disagree
- Strongly disagree
- Don’t know
  1. My partner doesn’t allow me to do some things
- Strongly agree
- Agree
- Disagree
- Strongly disagree
- Don’t know
  1. My partner makes most of the decisions
- Strongly agree
- Agree
- Disagree
- Strongly disagree
- Don’t know

1. When making decisions concerning your health, who has the most influence in the decisions that you make?

- Partner/s
- Peers/friends
- Parents
- Self
- Other, specify

**HIV and PrEP Knowledge**

1. Have you had a HIV test in the last 12 months?

- yes
- no

1. Which HIV prevention methods are you aware of? ***(Mark all that apply)***

- Condoms
- Pre-exposure prophylaxis
- HIV testing
- Treatment as prevention
- Abstinence
- None
- Other, specify: _______________

25a. Which HIV prevention methods have you ever used? ***(Mark all that apply****)*

- Condoms
- Pre-exposure prophylaxis (PrEP)
- HIV testing
- Treatment as prevention
- None
- Other, specify: _______________

1. Have you ever heard of oral Pre-exposure Prophylaxis (PrEP)?

- yes
- no
- Don’t know

If no, read; ***PrEP, or pre-exposure prophylaxis, is a daily pill to prevent HIV. PrEP is like the birth control pill taken to prevent pregnancy. PrEP must be taken every day to protect you from HIV just like the birth control pill is taken every day to prevent pregnancy.***

1. If yes, are you currently using PrEP?

- Yes
- No

30a. If no, would you be willing to use PrEP for HIV prevention?

- yes
- no
- Don’t know

1. Do you know anyone using PrEP?

- yes
- no
- Don’t know

1. Do you think pregnant and lactating women would be willing to use PrEP for HIV prevention?

- yes
- no
- Don’t know

1. What or who would influence their decision?

- Self/risk of HIV
- Partner/spouse
- Healthcare provider
- Other, please specify

**ACCEPTABILITY OF LONG-ACTING PRODUCTS AND RESEARCH PARTICIPATION**

Please read, **long-acting HIV prevention products are tools that do not require daily dosing. Instead, they can be inserted, injected, infused, or implanted in a person’s body from once or twice a month to once a year to provide sustained protection from acquiring HIV.**

1. How likely are you to participate in HIV prevention research involving long-acting HIV prevention products?

- Highly Likely
- Likely
- Not sure
- Less Likely
- Not likely

31a. If not likely, why would you not participate in research?

- I am worried about my safety
- I am worried about my child’s safety
- My partner will not allow me
- I do not like research

1. Do you think lactating women would be willing to use long-acting PrEP for HIV prevention?

- Yes
- No
- Don’t know

If no, why not, please clarify

1. Do you think pregnant women would be willing to use long-acting PrEP for HIV prevention

- Yes
- No
- Don’t know
- If no, why not, please clarify

1. Whom do you think would influence pregnant and lactating women participation in research of long-acting HIV prevention products?

- Spouse/ partners
- Friends/peers
- Parents
- Other, specify

1. In the future in addition to oral PrEP, other methods for long-acting HIV prevention may be available. Which long-acting product you would prefer? Please indicate whether [1. most preferred: 2 preferred, 3 moderately preferred, and 4 not preferred for each product]
   1. Vaginal ring

- Most preferred
- Preferred
- Moderately preferred
- Not preferred
  1. Injectable PrEP?
- Most preferred
- Preferred
- Moderately preferred
- Not preferred
  1. Implantable PrEP
- Most preferred
- Preferred
- Moderately preferred
- Not preferred
  1. Monthly Oral pill
- Most preferred
- Preferred
- Moderately preferred
- Not preferred
  1. Weekly Oral pill
- Most preferred
- Preferred
- Moderately preferred
- Not preferred

36a. Why is the most preferred long-acting method important to you? **(Tick all that apply)**

- Easy to use (you don’t have to worry of taking a pill)
- Dosage frequency
- Effective in preventing HIV
- Discreteness/privacy
- Accessibility
- Other, specify

1. What dosage frequency would you prefer for each PrEP formulation?
   1. Oral pills

- Only when you need it (On demand pill)
- Daily
- A monthly
- Weekly
- None
  1. Injection
- 2-monthly
- 3-monthly
- Every 6 months
- Yearly
- None
  1. Implant
- Monthly
- Every 3 months
- Every 6 months
- Yearly
- None
  1. Vaginal ring
- Monthly
- Yearly
- Every 3 months
- Every 6 months
- None

1. What type of packaging would you prefer for oral PrEP?

- Blister packs
- Bottles
- Packets/boxes
- Other, specify

1. Which kind of visit schedule for getting long-acting PrEP would work best for you?

- Monthly
- 3-Monthly
- 6-Montly
- Yearly

1. Where would you prefer to access long-acting HIV prevention products from? **(Tick all that apply).**

- Public health hospital outpatient clinic
- HIV Clinic (CCC)
- PrEP or HIV Prevention safe spaces
- Family Planning Clinic
- Maternal-child health clinic
- Hospital Pharmacy
- Hair Salons
- Chemist (community pharmacy)
- Other, specify

1. What concerns would you have when using Long-acting HIV prevention products?

- Safety (to the baby/pregnancy)
- Cost
- Potential intimate Partner violence
- Access
- No concerns
- Other specify

**HIV SELF TESTING AND PrEP USE**

1. Have you heard of HIV self-testing?

- Yes
- No

If not, read; **HIV self-testing is the use of special types of HIV-self test kits that women and men can use to test themselves for HIV. There are two types of HIV self-test kits, oral fluid based through oral swabbing, and blood based through finger prick.**

1. Which HIV self-testing method would you prefer?

- Oral Fluid based
- Blood-based
- None

1. Would you be fine in using HIV self-tests to test yourself for HIV instead of coming to the clinic at least some of the time while using long-acting PrEP products?

- Yes
- No
- Unsure

1. What concerns would you have when using HIV self-tests when using the long-acting PrEP products? **Tick all that apply**.

- Too hard to interpret results
- Worried about being seen with the HIV test kit
- Prefer to have health care professional interpret the test
- Worried about being able to use the self-test kit properly
- Concerned about the accuracy of the self-test kit
- Worried about potential of a positive result
- No concerns

**=END of Interview=**
